# Supplementary material for: Rectification and confinement of photokinetic bacteria in an optical feedback loop
Source: Nat Commun. 2022 May 18;13:2740. doi: 10.1038/s41467-022-30201-1 (PMC9117307; doi:10.1038/s41467-022-30201-1)
Supplement: Supplementary file 1 — Supplementary Information [file 41467_2022_30201_MOESM1_ESM.pdf]

Rectification and confinement of photokinetic bacteria in an optical feedback loop
Supplementary Information

Helena Massana-Cid,[1] Claudio Maggi,[1,2] Giacomo Frangipane,[3] and Roberto Di Leonardo[1,2,*]

[1] *Dipartimento di Fisica, Sapienza Università di Roma, Piazzale A. Moro 5, I-00185 Rome, Italy*
[2] *NANOTEC-CNR, Soft and Living Matter Laboratory, Institute of Nanotechnology, Piazzale A. Moro 5, I-00185 Rome, Italy*
[3] *Center for Life Nano- & Neuro-Science, Fondazione Istituto Italiano di Tecnologia (IIT), 00161 Rome, Italy*

(Dated: April 11, 2022)

Supplementary Movies

With the article there are three videoclips that supplement the figures in the main text.

- **Supplementary Movie 1** Photokinetic bacteria showing directed transport towards the right $\hat{n} = \hat{x}$. Dark-field microscope images of bacteria (red) is superimposed to the projected light pattern (green and black) with $R = 3.8$ µm $\Delta = 5.1$ µm and $\tau = 0.2$s The black background corresponds to minimum light intensity I_0 with associated cell velocity modulus v_0 and the green spots to maximum light intensity I_1 and v_1. White traces represent bacterial trajectories and are obtained by superimposing images acquired in the previous 2 s. The corresponding process is illustrated in Fig. 1 of the article. Playback speed is 4x.
- **Supplementary Movie 2** Confinement of bacteria in circles of different radii R_c. Feedback loop parameters are $\Delta = 1.8$ µm, $R = 2\mu$m, $\tau = 0.1s$, $v_1 = 10$µm s^{-1} and $v_0 = 5$µm s^{-1}. In the end, the feedback is turned off. The corresponding process is illustrated in Fig. 4a of the article. Playback speed is 10x and 40x, as indicated in the video.
- **Supplementary Movie 3** Confinement of bacteria by an optical feedback loop with parameters $\Delta = 3.3$µm, $R = 2.7$µm, $\tau = 0.1$s, $a = 91$µm, $v_1 = 10$µm s^{-1} and $v_0 = 5$µm s^{-1}. In the end, the feedback is turned off. The corresponding process is illustrated in Fig. 4b of the article. Playback speed is 100x and 10x, as indicated in the video.
- **Supplementary Movie 4** Splitting and merging of optically confined clouds of motile bacteria. We split an optically confined region of highly motile bacteria into two separate clouds and then merge them together. The corresponding process is illustrated in Fig. 5 of the article. Playback speed is 40x.

Supplementary Figure 1

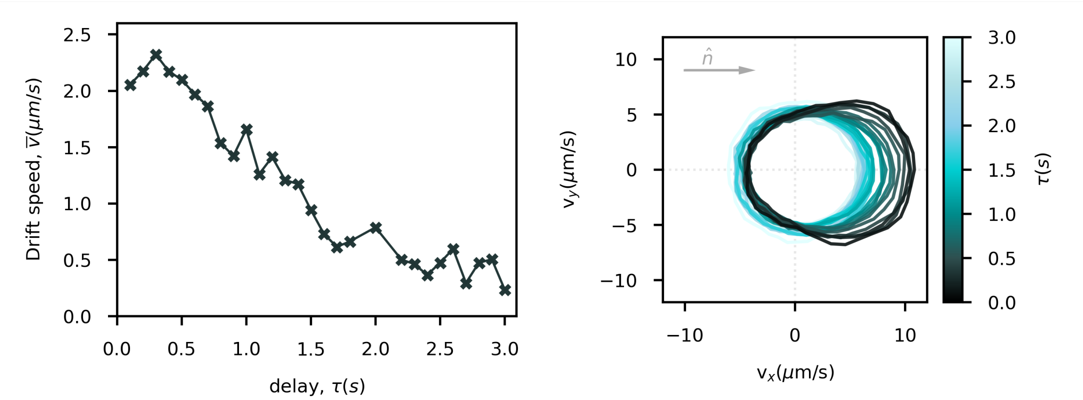

FIG. 1. **Dependency of the drift speed and on the time delay τ and corresponding polar plots**. In these experiments, $R = 3.8$ µm, $v_1 = 10$ µm s^{-1}, $v_0 = 3.5$ µm s^{-1}.

Supplementary Note 1: RnT model with feedback

To check in detail the theoretical predictions we simulate the "Run and Tumble" (RnT) model with direction-dependent speed. In these simulations RnT particles move in a squared box extending between $\pm L/2$ both along x and y (periodic boundary conditions apply). In the case where the feedback is applied only along the x-axis, particles at $|x| > a$ move at

speed v_0 when pointing away from $x=0$ (while moving at speed $v_1 > v_0$ when pointing towards $x=0$). If $|x| < a$ particles always move at maximum speed v_1. We use the experimental values for the speeds $v_0 = 5$ µm s^{-1}, $v_1 = 10$ µm s^{-1}, and for the tumbling rate $\lambda = 0.75$ Hz. Since these parameters correspond to a decay length $\ell \approx 20$ µm we set the simulation box size to $L = 750$ µm ($\gg \ell$).

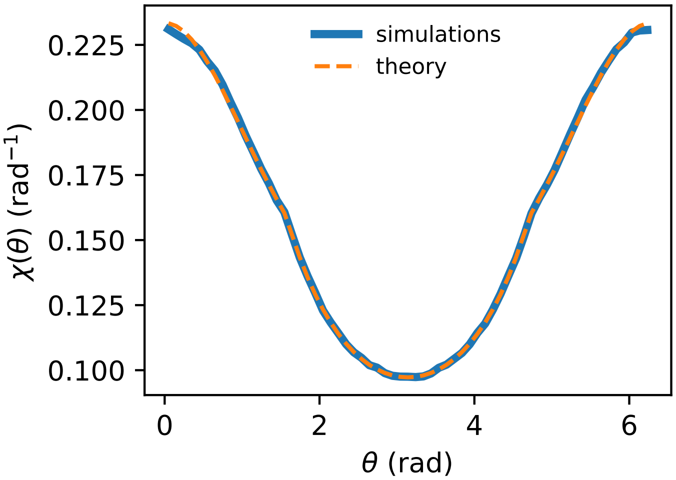

FIG. 2. The angular probability density $\chi(\theta)$ obtained in simulations is represented by the full thick line and this is well described by the theoretical prediction $\chi(\theta) = [(2\pi)(1 - k\,v(\theta)/\lambda)]^{-1}$ (dashed line).

After reaching the stationary state the density profile shows a clear exponential form for $|x| > a$ as shown Fig. 3 of the main text (i.e. $\rho(x) \propto e^{-k|x-a|}$) as predicted by the theory. Here we show (see Fig. 2) that also the probability density $\chi(\theta)$ of the particles orientation θ (full line) agrees with theory. To do this computed only for particles that are at least one persistence length away from the border (i.e. for $|x| > a + v_1/\lambda$). We recall that the angular theoretical distribution is given by:

$$\chi(\theta) = \frac{1}{2\pi} \frac{1}{1 - kv(\theta)\cos\theta/\lambda} \tag{1}$$

which is plotted as a dashed curve in In Fig. 2 (where $v(\theta) = v_0$ if $|\theta| < \pi/2$ and $v(\theta) = v_1$ otherwise). As described in the main text the value of k is obtained by imposing normalization of the angular distribution:

$$1 = \int_0^{2\pi} \chi(\theta) d\theta = \int_0^{2\pi} \frac{1}{2\pi} \frac{1}{1 - hv(\theta)\cos\theta} d\theta = \frac{1}{\pi}\left[\int_0^{\pi/2} \frac{1}{1 - hv_1\cos\theta} d\theta + \int_{\pi/2}^{\pi} \frac{1}{1 - hv_0\cos\theta} d\theta\right] \tag{2}$$

where we introduced the variable $h = k/\lambda$. After some simple manipulation we get:

$$\int_0^{\pi/2} \frac{1}{1 - hv_0\cos\theta} d\theta + \int_0^{\pi/2} \frac{1}{1 + hv_1\cos\theta} d\theta - \int_0^{\pi/2} 2 d\theta = 0 \tag{3}$$

which can be rewritten as:

$$\int_0^{\pi/2} \frac{2h^2 v_0 v_1 \cos^2\theta - h(v_1 - v_0)\cos\theta}{1 + h(v_1 - v_0)\cos\theta - h^2 v_0 v_1 \cos^2\theta} d\theta = 0 \tag{4}$$

Now we numerically find that for typical parameters value the denominator is a slowly varying function of θ and a very good estimate for h can be found by simply imposing that the integral of the numerator vanishes:

$$\int_0^{\pi/2} 2h^2 v_0 v_1 \cos^2\theta - h(v_1 - v_0)\cos\theta = \int_0^{\pi/2} 2h^2 v_0 v_1 \cos^2\theta - h(v_1 - v_0)\cos\theta = \frac{\pi}{2} h^2 v_1 v0 - h(v_1 - v_0) = 0 \tag{5}$$

from which we obtain:

$$k = h\lambda = \frac{2\lambda}{\pi}\frac{v_1 - v_0}{v_1 v_0} \tag{6}$$

Supplementary Note 2: Primers for atp operon deletion using pKD3 as template

1. GCGATCGCTTTGGCCAGTTCCGCAGACGCCTGAGCGTAATCTACGTCGCCGTGTAGGCTGGAGCTGCTTC
2. CGGCATACCTCGAAGGGAGCAGGAGTGAAAAACGTGATGTCTGTGTCGCTATGGGAATTAGCCATGGTCC

* roberto.dileonardo@uniroma1.it
