# Supplementary material for: Rectification and confinement of photokinetic bacteria in an optical feedback loop
Source: Nat Commun. 2022 May 18;13:2740. doi: 10.1038/s41467-022-30201-1 (PMC9117307; doi:10.1038/s41467-022-30201-1)
Supplement: Supplementary file 3 — Description to Supplementary Information [file 41467_2022_30201_MOESM3_ESM.pdf]

# Description of Additional Supplementary Information

- **Supplementary Movie 1** Photokinetic bacteria showing directed transport towards the right $\hat{n} = \hat{x}$. Dark-field microscope images of bacteria (red) is superimposed to the projected light pattern (green and black) with $R = 3.8$ µm $\Delta = 5.1$ µm and $\tau = 0.2$s The black background corresponds to minimum light intensity $I_0$ with associated cell velocity modulus $v_0$ and the green spots to maximum light intensity $I_1$ and $v_1$. White traces represent bacterial trajectories and are obtained by superimposing images acquired in the previous $2$ s. The corresponding process is illustrated in Fig. 1 of the article. Playback speed is 4x.

- **Supplementary Movie 2** Confinement of bacteria in circles of different radii $R_c$. Feedback loop parameters are $\Delta = 1.8$ µm, $R = 2\mu$m, $\tau = 0.1s$, $v_1 = 10$µm s$^{-1}$ and $v_0 = 5$µm s$^{-1}$. In the end, the feedback is turned off. The corresponding process is illustrated in Fig. 4a of the article. Playback speed is 10x and 40x, as indicated in the video.

- **Supplementary Movie 3** Confinement of bacteria by an optical feedback loop with parameters $\Delta = 3.3$µm, $R = 2.7$µm, $\tau = 0.1$s, $a = 91$µm, $v_1 = 10$µm s$^{-1}$ and $v_0 = 5$µm s$^{-1}$. In the end, the feedback is turned off. The corresponding process is illustrated in Fig. 4b of the article. Playback speed is 100x and 10x, as indicated in the video.

- **Supplementary Movie 4** Splitting and merging of optically confined clouds of motile bacteria. We split an optically confined region of highly motile bacteria into two separate clouds and then merge them together. The corresponding process is illustrated in Fig. 5 of the article. Playback speed is 40x.
